# The impact of hotel robots’ service quality on continuance intention: the moderating effect of personal innovation

**DOI:** 10.3389/frobt.2025.1667123

**Published:** 2025-09-10

**Authors:** Yaru Shi, Weifang Zhan, Chengpeng Lin

**Affiliations:** 1 Department of Hotel & Convention, Honam University, Gwangju, Republic of Korea; 2 Department of Business Administration, Chonnam National University, Gwangju, Republic of Korea

**Keywords:** reliability, assurance, entertainment, anthropomorphism, tangibles, continuance intention, personal innovation

## Abstract

As service robots are increasingly integrated into the hotel industry to enhance operational efficiency and customer experience, understanding consumers’ responses to robotic services has become a critical research agenda. However, empirical evidence on how customers evaluate the service quality of hotel robots and how these evaluations influence their continuance intention remains limited. Drawing on the SERVQUAL framework, this study redefines service quality in the context of AI-powered hotel robots through five dimensions: reliability, assurance, entertainment, anthropomorphism, and tangibles. Furthermore, the study explores the moderating role of personal innovativeness in the relationship between perceived service quality and continuance intention. Data were collected via an online survey from 400 Generation Z consumers in China who had prior experience with item-delivery robots in hotel settings. The results indicate that assurance, entertainment, anthropomorphism, and tangibles have significant positive effects on continuance intention, while reliability does not show a statistically significant impact. Moreover, personal innovativeness significantly moderates the effects of certain service quality dimensions, suggesting that individual differences in technology readiness shape consumer reactions to robotic services. This study contributes to the literature by extending traditional service quality theory into the domain of human–robot interaction and by highlighting the nuanced mechanisms through which robot-specific service attributes influence user loyalty. Practical implications are offered for hotel managers seeking to optimize robot deployment strategies and improve guest engagement in technology-enhanced service environments.

## 1 Introduction

Hotels operate in a service-intensive industry. With the advent of the Fourth Industrial Revolution, intelligent service robots have been developing rapidly (Baraka and Veloso, 2018). Currently, service robots are widely deployed in various hotel operations, including reception, food delivery, and room cleaning. Digital technologies such as artificial intelligence (AI), the Internet of Things (IoT), and cloud computing are advancing at an unprecedented pace, reshaping service processes and altering customer expectations and experiences (Wirtz et al., 2018; Meyer-Waarden et al., 2020). The potential for robot services to substitute human labor is attracting increasing attention, with some hotels already integrating robots into daily operations (Mende et al., 2019; Murphy et al., 2021). These robots can deliver more efficient and accurate services, thereby reducing labor costs and enhancing customer experience (Zhong et al., 2020). Additionally, an aging global population and rising labor costs further drive automation demand in the hotel industry (Tang and Lee, 2019).

In response to this technological shift, academic interest in service robots within hospitality and tourism has grown. Prior research has focused on customers’ attitudes toward service robots (Ivanov et al., 2018; Lin and Mattila, 2021; Zhong et al., 2020; Jia et al., 2021; Ayyildiz et al., 2022; Kim et al., 2021; Lu et al., 2019), quality of robot services as perceived by customers (Kharub et al., 2021), adoption behaviors (Lu et al., 2019; Ozturk et al., 2023), the role of service robots in hospitality operations (Ivanov et al., 2019; Koo et al., 2021; Fuentes Moraleda et al., 2020), and customer experiences with service robots (Tung and Law, 2017). Despite this growing interest, customer perceptions of service robots remain complex and somewhat inconsistent, necessitating further investigation (Lu et al., 2019).

However, most existing studies concentrate on early acceptance, novelty effects, or interaction mechanisms, with limited attention to how various service quality dimensions influence long-term customer behaviors such as continuance intention. In particular, the role of service quality has been underexplored in the context of hospitality robots. Service quality is generally defined as customers’ overall evaluation of a service provider’s excellence or superiority (Parasuraman et al., 1988). The original SERVQUAL framework identifies five dimensions—tangibles, reliability, responsiveness, assurance, and empathy. While tangibles, reliability, and assurance remain relevant to the hotel robot context, the constructs of responsiveness and empathy require adaptation. In human-to-human service, responsiveness refers to the promptness of service delivery and empathy reflects the provider’s ability to understand and care about customers’ needs (Parasuraman et al., 1988). However, robots lack genuine emotions and human cognitive empathy, and their “responsiveness” is primarily determined by pre-programmed speed and automation capabilities rather than interpersonal sensitivity. To better capture robot-specific service attributes, this study replaces “responsiveness” and “empathy” with entertainment and anthropomorphism. Entertainment refers to the extent to which interacting with the robot is enjoyable, engaging, and stimulating for customers, which has been shown to enhance satisfaction and technology acceptance in hospitality settings (Tung and Au, 2018; Tussyadiah and Park, 2018). Anthropomorphism reflects the degree to which a robot exhibits human-like appearance, expressions, or behaviors, fostering social presence and trust (Epley et al., 2007; Kim et al., 2021). Prior research in service robotics suggests that these two dimensions are particularly salient in shaping customer experience because robots often serve not only functional roles but also hedonic and relational ones (Wirtz et al., 2018; de Kervenoael et al., 2020). By introducing these constructs, the adapted model better reflects the unique interactional dynamics between customers and hotel robots, aligning aligning the measurement framework with the technological and experiential nature of robot-delivered services.

Furthermore, individual differences in consumer behavior, such as personal innovativeness—defined as an individual’s propensity to embrace and experiment with new technologies (Agarwal and Prasad, 1998)—have rarely been examined in the context of robot services. Prior research suggests that younger, more innovative individuals are more likely to trust and adopt new technologies (Arfi et al., 2021), yet the moderating role of personal innovativeness on the relationship between robot service quality and continuance intention remains underexamined. This study therefore investigates personal innovativeness as a moderating variable. Additionally, it focuses on Generation Z consumers, who are digital natives exposed to smart technologies and internet connectivity from an early age. Gen Z typically demonstrates greater openness to technological innovation, higher expectations for digital convenience, and stronger preferences for personalized, interactive experiences. Understanding their attitudes toward service robots is critical as they become a dominant consumer segment in hospitality. Based on these considerations, this research explores how multiple dimensions of hotel robot service quality—including reliability, assurance, entertainment, anthropomorphism, and tangibles—affect customers’ continuance intention. It also examines the moderating effect of personal innovativeness on this relationship. The findings aim to contribute theoretically to the hospitality robot literature and offer practical guidance for improving robot-based service strategies in hotels.

The remainder of this paper is organized as follows: Section 2 presents the literature review and hypothesis development; Section 3 details the research methodology; Section 4 reports the results of hypothesis testing; Section 5 discusses findings, limitations, and future research directions.

## 2 Literature review and hypotheses development

### 2.1 Service robots in hotel

In recent years, integrating robots into hotel services has become a significant component of the broader digital transformation in the hospitality industry. Robotics technology has had a profound impact on customers, businesses, and communities in the hotel industry (Ivanov and Webster, 2021). The industry’s operations are increasingly dependent on technologies that can effectively respond to customer needs by enhancing both service experiences and operational efficiency (Gretzel, 2021). Prior studies have highlighted several benefits of adopting service robots in hospitality settings, including reduced operational costs (Ivanov et al., 2020), improved efficiency (Ivanov et al., 2018), enhanced customer experience (Ivanov and Webster, 2021; Tung and Law, 2017), and strengthened business competitiveness (Ivanov and Webster, 2018). Although the adoption of service robots can lead to improvements in operational efficiency and customer experience, it requires careful and strategic implementation (Lee, 2024). Previous research has also highlighted the various challenges that hospitality and tourism firms encounter when integrating service automation through service robots (Ivanov et al., 2017). For instance, when service errors occur, customers still tend to prefer human employees to handle them (Lin and Mattila, 2021).

Hotels have increasingly introduced robots to perform various tasks such as food delivery, concierge services, and automatic check-in, which have attracted growing attention from both practitioners and academics. For example, the Hilton Hotel Group introduced the robot Connie in collaboration with IBM to assist guests with information about local attractions and services (Tussyadiah and Park, 2018). Japan’s Henn-na Hotel has implemented robotic receptionists and concierge robots to provide intelligent hotel services (Choi et al., 2019), while the Royal Caribbean cruise ship Quantum features a robotic arm functioning as a bartender (Chan and Tung, 2019). Upon arrival, it automatically opens its lid, allowing guests to retrieve their ordered items such as food or amenities (Choi et al., 2019). These examples underscore the expanding use of service robots across various hospitality contexts.

Despite the increasing deployment of service robots, existing studies primarily focus on customers’ acceptance and usage intentions based on theoretical models such as the Technology Acceptance Model (TAM) and the Theory of Planned Behavior (TPB). Fuentes Moraleda et al. (2020) developed a hotel-specific service robot acceptance model (SRAM) to investigate human-robot interaction. Zhong et al. (2021) integrated perceived value into their acceptance model, showing how it influences customers’ recognition of robot service. Jia et al. (2021) highlighted the importance of anthropomorphism in shaping tourist behavior, while Huang et al. (2021) examined how perceived identity threats and anthropomorphic features influence negative attitudes toward hotel robots. Additionally, Mariani and Borghi (2021) analyzed AI-related text reviews to evaluate customer perceptions, and Fu et al. (2022) addressed resistance to robot technology in hotels using the Robot Usage Resistance Model. Overall, these studies help deeply understand the acceptance of hotel service robots, their impact on customer behavior and the factors influencing their adoption in various contexts.

### 2.2 Service quality of robots

Service quality has long been recognized as a foundational concept in service marketing and customer satisfaction research (Parasuraman et al., 1988). Among the service quality models, SERVQUAL (Parasuraman et al., 1988) is the most widely adopted framework and has been validated across a wide range of contexts such as airlines (Shah et al., 2020), healthcare (Singh and Prasher, 2019), and tourism (Chand, 2010). SERVQUAL originally proposed five dimensions: reliability, assurance, tangibility, empathy, and responsiveness. These dimensions were designed to evaluate the gap between customers’ expectations and perceptions of service quality.

As AI and robotics are increasingly incorporated into the service industry, scholars have turned their attention to robotic service quality. However, the traditional SERVQUAL model has been criticized for its inadequacy in addressing the unique characteristics of robotic services (Prentice and Nguyen, 2021). For example, it emphasizes functional aspects (e.g., responsiveness, empathy) but overlooks technical outcomes, which are essential in robot-mediated service interactions. Several scholars have attempted to adapt or expand the SERVQUAL model to evaluate robotic service quality. Prentice and Nguyen (2021) introduced a four-dimensional scale: automation, personalization, precision, and efficiency, specifically targeting robotic service experiences. In hospitality, Choi et al. (2019) validated a model distinguishing between interaction quality, outcome quality, and physical service environment. Interaction quality evaluates customer-robot interactions during the service process, including how effective and dependable robots are. Outcome quality reflects the technical performance after service delivery, such as solving problems or providing personalized services. Physical service environment includes spatial layout and design accommodating robots. This study adopts three original SERVQUAL dimensions - reliability, assurance, and tangibles - (Parasuraman et al., 1988) while incorporating two additional dimensions relevant to the hotel robot context: entertainment and anthropomorphism (Schodde et al., 2017; Jia et al., 2021; Huang et al., 2021). Entertainment is included as a key driver in the adoption of social robots (Schodde et al., 2017), Anthropomorphism and entertainment value play critical roles in customer satisfaction and acceptance of service robots (Jia et al., 2021; Huang et al., 2021). Robots with human-like tangibles and entertaining features can enhance emotional engagement and trust, especially in hospitality contexts where emotional responses are significant. In summary, this study retains the basic structure of the SERVQUAL model and appropriately revises the measurement framework by incorporating contextual characteristics of hotel service robots. As a result, five key dimensions of service quality are proposed: reliability, assurance, entertainment, anthropomorphism, and tangibles.

In the field of human-computer interaction, reliability was defined as the ability of robots to consistently deliver on their service commitments (Chiang and Trimi, 2020). When using robots to provide information, reliability was about the accuracy of the information provided (Xifei and Jin, 2015). This concept was consistent with the traditional service quality framework put forward by Parasuraman et al. (1988), which put emphasis on the accurate performance of the service. Empirical research further stresses reliability is crucial in service environments. For example, in the Robot Cafe study, customers thought highly of the reliability of service robots, which in turn increased their willingness to engage with robots repeatedly (Morita et al., 2020). This highlights how reliability directly affects customers’ continuance intention in the robots. First, assurance referred to the customer’s view of the quality of product or service, reflecting the service robot’s expertise, courtesy, customer orientation and the sense of security it provides (Varki and Colgate, 2001). In the context of social robots, with focus on the robots’ ability to lead to customer trust, assurance mattered in maintaining and enhancing the service quality in the hotel and tourism industries (Ivkov et al., 2020). In certain cultural backgrounds, this was identified as the most crucial in the service quality (Raajpoot, 2004). Second, entertainment refers to the intrinsic enjoyment and pleasure derived from service interactions. In the hotel industry, service robots can not only deliver basic information but also provide engaging and enjoyable experiences for guests (Mele et al., 2020). Perceived entertainment is defined as the degree of hedonic satisfaction experienced during service usage, reflecting the user’s cognitive and affective engagement. Lin and Hsieh (2011) argued that entertainment mattered in the primary motivation for customers to use hedonic systems. Similarly, Conti et al. (2017) found that perceived entertainment in robotics research significantly increased their willingness to interact with robots by impacting users’ intentions to use robots. According to the previous research, users assessed both the functional use of technology and the entertainment values provide by the technology (Kim and Forsythe, 2008). Social robots enjoyed entertainment functions for a long time (Anselmsson, 2016), such as their physical tangibles, facial expressions and tone, as well as controlled dynamic movements. When robots were customized according to specific environment, they may also have temporary entertainment functions (Elmashhara and Soares, 2020). Fourth, anthropomorphic robots resemble humans in terms of function and structure (Phillips et al., 2018). In Anthropomorphics, there was the tendency to attribute humanoid characteristics and motivations to the real or imagined behavior of non-human agents (Fink, 2012; Morewedge et al., 2007). Service robots can be classified into mechanical robots based on the level of anthropomorphism in their physical tangibles, just like human robots (Walters et al., 2008). According to Wan and Aggarwal (2015), anthropomorphic perception in robots, including intentional and emotional attribution, was closely related to their task performance. They considered that anthropomorphic service robots can be an effective strategy to enhance consumer preferences. Krach et al. (2008) reported that consumers experienced more fun when interacting with robots that resembled humans. Finally, tangibles refer to the physical facilities, equipment, the appearance of personnel, and the appearance of written or digital materials related to the service provider (Parasuraman et al., 1988). In the service robot environment, tangibles not only include traditional physical facilities and equipment but also encompass the robot’s exterior design, the aesthetics and functionality of the interactive interface, as well as the overall visual and functional experience of related hardware devices in the service setting. These factors collectively influence customers’ perceptions of robot service quality.

### 2.3 Service quality and continuance intention

Continuance intention refers to an individual’s willingness to persist in using a product or service over time. According to the Theory of Planned Behavior (Ajzen, 1991), intention is the most immediate predictor of actual behavior and is influenced by an individual’s attitude toward the behavior, subjective norms, and perceived behavioral control. In the context of technology-based services, Bhattacherjee (2001) emphasized that continuance intention is shaped by prior usage experiences and the confirmation of expectations, reflecting users’ motivation to maintain ongoing usage. Furthermore, Kuo et al. (2009) noted that continuance intention also encompasses consumers’ willingness to recommend or share their experiences, which indicates a high level of satisfaction and loyalty.

In service research, service quality has been widely recognized as a primary determinant of both customer satisfaction and continuance intention (Zeithaml et al., 1996). High service quality enhances users’ trust, satisfaction, and emotional connection, all of which positively contribute to their intention to continue using the service (McDougall and Levesque, 2000; Srivastava and Vishnani, 2021). Empirical studies in various sectors, including transportation and online platforms, have confirmed the positive effect of perceived service quality on continued use (Wang et al., 2020; Liu, 2020).

However, in the emerging field of hotel service robots, research examining the direct influence of specific service quality dimensions—such as reliability, assurance, entertainment, anthropomorphism, and tangibles—on continuance intention remains limited. Given the novelty of robotic services in hospitality and the distinct characteristics that differentiate them from traditional human-delivered services, existing theoretical frameworks and empirical findings may not fully capture these dynamics (Prentice and Nguyen, 2021; Jia et al., 2021). This highlights the need for more focused studies that explore how robot-specific service quality attributes impact users’ ongoing engagement and loyalty. In the specific context of hotel service robots, the relationship between service quality and continuance intention is more complex. Unlike traditional human-based services, robot-delivered services often lack empathy and natural interpersonal interaction, which may influence users’ perceptions and trust formation (Prentice and Nguyen, 2021). However, when service robots demonstrate high levels of functional reliability, anthropomorphism, and entertainment value, they can provide memorable experiences that reduce user resistance and foster emotional acceptance (Jia et al., 2021; Huang et al., 2021). These robot-specific quality attributes can therefore influence not only customer satisfaction but also behavioral loyalty and continuance intention.

Based on the above theoretical foundation and context-specific considerations, this study proposes that the quality of hotel robot services has a significant positive effect on customers’ continuance intention. The hypotheses are formulated as follows:

H1: The service quality positively influences continuance intention.
H1a: The reliability of service quality positively influences continuance intention.
H1b: The assurance of service quality positively influences continuance intention.
H1c: The entertainment of service quality positively influences continuance intention.
H1d: The anthropomorphism of service quality positively influences continuance intention.
H1e: The tangibles of service quality positively influences continuance intention.

### 2.4 The moderating effect of personal innovation

Personal innovativeness refers to the degree to which an individual is willing to adopt and try new technologies earlier than others within a social system (Rogers, 2003). It reflects openness to novel experiences and a desire for stimulation, particularly in the context of emerging technologies (Agarwal and Prasad, 1998; Rogers et al., 2014). Agarwal and Prasad (1997) defined personal innovativeness as an individual’s willingness to engage with new information technologies, serving as a key factor distinguishing early adopters from non-adopters. From the perspective of the Diffusion of Innovation Theory (Rogers, 2003), individuals high in innovativeness are more likely to overcome uncertainties and perceived risks associated with emerging technologies, leading to faster and more sustained adoption. Furthermore, the Unified Theory of Acceptance and Use of Technology (UTAUT) model highlights personal traits like innovativeness as key moderators that influence the strength of relationships between technological attributes (e.g., performance expectancy, effort expectancy) and behavioral intentions (Venkatesh et al., 2003).

Within the hospitality industry, particularly in the context of novel service modalities such as service robots, highly innovative consumers tend to have a more positive evaluation of robotic service quality and exhibit greater willingness to continue usage (Hsu and Chiu, 2004). These consumers typically adapt more readily to technology-mediated interactions and derive enhanced satisfaction from the novelty and efficiency these services provide (Davenport and Ronanki, 2018). Empirical studies underscore the moderating role of personal innovativeness on the link between service quality and usage intentions. For instance, Goldsmith and Hofacker (1991) demonstrated that individuals with higher innovativeness exhibit greater acceptance of new technologies. Wan and Aggarwal (2015) found that personal innovativeness strengthened the positive influence of anthropomorphic robot attributes on continuance intention, suggesting that innovative users are more responsive to human-like features in robots. Similarly, Hussain et al. (2019) identified that innovation-oriented users showed higher sensitivity to playful and aesthetic design elements, which are crucial hedonic dimensions of robotic services. These findings align with the notion that personal innovativeness not only fosters initial adoption but also sustains long-term engagement by buffering potential negative perceptions and enhancing emotional acceptance. In the specific context of hotel service robots, which present unique challenges such as limited empathy and natural interaction compared to human staff (Prentice and Nguyen, 2021), personal innovativeness may play a pivotal role in moderating how customers perceive and respond to diverse service quality dimensions—including reliability, assurance, entertainment, anthropomorphism, and tangibles. Innovative consumers may be more forgiving of service shortcomings or may derive greater pleasure and trust from robot features that non-innovators might undervalue, thus amplifying the impact of service quality on continuance intention.

Therefore, integrating insights from technology acceptance theories and hospitality service literature, this study posits that personal innovativeness functions as a significant moderator in the relationship between hotel robot service quality and customers’ continuance intention. This conceptualization enhances the explanatory power of the model by accounting for individual differences in technology readiness and behavioral adaptation. Based on the above theoretical and empirical rationale, the following hypotheses are proposed:

H2: Personal innovation moderates the relation between service quality and continuance intention, such that the relationship is stronger for individuals with higher personal innovativeness.
H2a: Personal innovation moderates the relation between service quality’s reliability and continuance intention.
H2b: Personal innovation moderates the relation between service quality’s assurance and continuance intention.
H2c: Personal innovation moderates the relation between service quality’s entertainment and continuance intention.
H2d: Personal innovation moderates the relation between service quality’s anthropomorphic and continuance intention.
H2e: Personal innovation moderates the relation between service quality’s tangibles and continuance intention.

**FIGURE 1:**
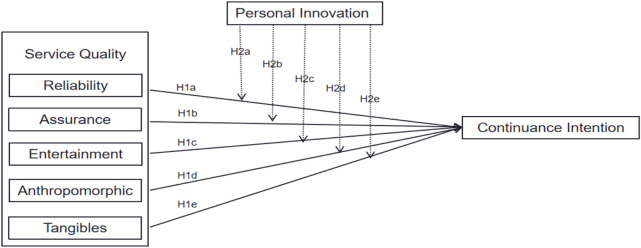
Conceptual model.

In summary, this study proposes a conceptual model. As shown in Figure 1, the model outlines the hypothesized relationships.

## 3 Research methodology

### 3.1 Measurement instrument

According to previous studies, the questionnaire was adapted to fit the context of hotel item delivery robots. All items were measured using a 5-point Likert scale ranging from 1 (“strongly disagree”) to 5 (“strongly agree”), ensuring clarity, logical arrangement, and content validity. The measurement covered seven constructs: Reliability (3 items, measuring the accuracy of task execution and the protection of customers’ personal and purchase information), Assurance (4 items, assessing the degree of confidence customers have in the robot’s service competence, support, and quality outcomes), Entertainment (3 items, evaluating the freshness, curiosity, and enjoyment experienced when interacting with the robot), Anthropomorphism (4 items, capturing human-like attributes such as consciousness, friendliness, emotional expression, and human-like responsiveness), Tangibles (3 items, reflecting the robot’s size appropriateness, attractiveness of appearance, and functional storage space), Continuance Intention (4 items, measuring the willingness to continue using, recommend to others, increase usage frequency, and prioritize the robot service), and Personal Innovation (3 items, assessing openness to new technologies and the tendency to be among the first to try them). The service quality items, continuance intention items, and personal innovation items were adapted from Kharub et al. (2021), Jo (2022), and Sowmya et al. (2024), respectively. A translation–back translation procedure was conducted to ensure linguistic equivalence between the original English items and the Chinese version. The questionnaire was pre-tested with 30 participants from the target population to ensure cultural appropriateness, clarity, and content validity.

### 3.2 Subject and data collection

By conducting a questionnaire survey on Generation Z customers in China who have used hotel robot services, this study has explored how the robot service quality in hotels influenced continuance intention and examined the moderating effect of personal innovation. The questionnaire was distributed online through the Questionnaire Star platform from October 1 to November 1, 2024, covering all provinces and major cities in mainland China to ensure the geographical diversity of the samples. The survey links are disseminated online through mainstream social media platforms such as WeChat, Weibo, and Douyin. To enhance sample representativeness, this study combined the random push function of the questionnaire star platform with stratified random sampling strategies, distributing samples by region and gender ratio to reduce sample bias. A total of 450 questionnaires were collected. Before participating in the survey, all participants were informed about the study’s purpose, voluntary nature, and their right to withdraw at any time without penalty. Participants provided informed consent by checking a consent box on the platform before proceeding to the questionnaire. This process ensured that participants’ involvement was voluntary and based on their understanding of the study. Besides, the screening questions in the survey were included to ensure better reliability and consistency of the online data. There were questions in the questionnaire, for example, have you used a hotel delivery robot within the past year? If their answer is yes, they can continue to fill in the questions. The subjects can only move to the next page after answering all the questions on the previous page. The criteria for determining invalid questionnaires include completion time of less than 3 min, continuous straight-line responses, logical contradictions, or missing values exceeding 10%. After discarding ineffective replies, 400 useful data were utilized for further analysis. The statistical software such as SPSS and Amos was employed for data screening and coding, and the descriptive statistics, reliability analysis, confirmatory factor analysis, moderation analysis and regression analysis were conducted to validate the hypothesis model. Table1 exhibits the demographic information of subjects in the final sample.

**TABLE 1:** Profile of respondents.

Demographics	Item	Subjects(N = 400)	
		Frequency	Percentage
Gender	Male	182	45.5%
	Female	218	54.5%
Age	18–24	168	41.7%
	24–30	233	58.2%
Career	Student	55	13.7%
	Production technicians	78	19.5%
	Professional office staff	95	23.7%
	Sales and service industry	105	26.2%
	Other	67	16.7%
Monthly income	410–690$	139	34.7%
	690–1,100$	127	31.7%
	>1,100$	134	33.5%
Education	<High School	20	5%
	Vocational school	119	29.7%
	bachelor	191	47.7%
	Master or higher	70	17.5%

### 3.3 Measurement model

Both the reliability and effectiveness of the measurement model were evaluated in this research. To assess reliability, it studied Cronbach’s alpha and composite reliability (CR). If estimates of Cronbach’s alpha and CR exceeded 0.7, the reliability would be realized (Nunnally, 1978). Hair et al. (2021) suggested a minimum requirement value of 0.70 for roh3. As displayed in Table 2, all values of the construct (Cronbach’s alpha, CR) were greater than 0.8, much higher than the expected threshold, which confirmed the effectiveness of the measurement model. The Average Variance Extraction (AVE) and the factor loadings associated with each structure were calculated to verify the convergence effectiveness. The range of AVE values was 0.558–0.744, which was greater than the recommended threshold of 0.5 (Fornell and Larcker, 1981; Tavakol and Dennick, 2011). The factor loadings ranged from 0.694 to 0.908, with statistical significance at the p ¼ 0.001 level, which revealed the satisfactory convergent validity (Bagozzi et al., 1991). The fit indices of the structural model in this study showed a chi-square value of 804.221 with 231 degrees of freedom, resulting in a chi-square/df ratio of 3.481, which meets the basic requirements for model fit. The CFI was 0.898, close to the ideal threshold of 0.90, and the RMSEA was 0.079, slightly below the acceptable standard of 0.08, indicating a good model fit. Two criteria were utilized to test the discriminant validity. Firstly, this study applied the Fornell Larcker criteria. Compared to other structures, the score on the diagonal within the same structure should be higher. According to Table 3, the square root of AVE for each construct was greater than the correlation value for that column or row, indicating the uniqueness of the studied structure.

**TABLE 2:** Scale reliabilities.

Constructs	Statement	Label	Loadings	Cronbach α	CR	AVE
Reliability	I believe hotel delivery robots can keep guests’ personal information confidential	REL1	0.852	0.871	0.876	0.703
	I believe hotel delivery robots can securely manage personal and purchase information	REL2	0.899			
	The business tasks of hotel delivery robots are error-free	REL3	0.758			
Assurance	Hotel delivery robots have strong task execution capabilities	ASS1	0.740	0.835	0.835	0.558
	The service support of hotel delivery robots is reliable	ASS2	0.722			
	Hotel delivery robots deliver high-level service outcomes	ASS3	0.715			
	Hotel delivery robots provide efficient and accurate services	ASS4	0.808			
Entertainment	Using hotel delivery robots feels refreshing	ENT1	0.792	0.818	0.825	0.611
	Using hotel delivery robots arouses curiosity	ENT2	0.826			
	The service process of hotel delivery robots is very enjoyable	ENT3	0.724			
Anthropomor-phism	Hotel delivery robots sometimes seem to have human-like awareness	ANTH1	0.709	0.844	0.845	0.578
	I think the task execution of hotel delivery robots is similar to that of humans	ANTH2	0.808			
	Hotel delivery robots sometimes make people feel friendly	ANTH3	0.767			
	Hotel delivery robots can express emotions and respond sensitively	ANTH4	0.753			
Tangibles	The size of hotel delivery robots is appropriate	TAN1	0.822	0.888	0.897	0.744
	The exterior design of hotel delivery robots is attractive	TAN2	0.883			
	Hotel delivery robots have appropriate storage space	TAN3	0.881			
Personal Innovation	I like trying new products	PI1	0.694	0.805	0.827	0.618
	When I hear about new information technology, I want to try it	PI2	0.908			
	Among my peers, I am usually the first to try new products	PI3	0.741			
Continuance intention	I will continue to use hotel delivery robots in the future	CI1	0.758	0.868	0.870	0.626
	I will actively recommend hotel delivery robots to others	CI2	0.814			
	I am willing to increase the frequency of using hotel delivery robots	CI3	0.816			
	I will give priority to using hotel delivery robots	CI4	0.775			

**TABLE 3:** Correlation matrix and discriminant assessment.

Constructs	REL	ASS	ENT	ANTH	TAN	PI	CI
REL	0.838						
ASS	0.305	0.747					
ENT	0.288	0.667	0.782				
ANTH	0.259	0.656	0.477	0.760			
TAN	0.093	0.502	0.272	0.412	0.862		
PI	0.052	0.260	0.056	0.289	0.662	0.786	
CI	0.212	0.669	0.604	0.598	0.441	0.063	0.791

## 4 Results of hypotheses testing

### 4.1 The influence of service quality on continuance intention

To examine the influence of service quality on continuance intention, a structural equation modeling (SEM) analysis was conducted, as shown in Table 4. The model did not include control variables. The findings revealed among the five dimensions of service quality, reliability did not influence continuance intention obviously (β = −0.009, C.R. = -0.24, p = 0.811), assuming H1a was rejected. However, assurance, entertainment, anthropomorphism and tangibles had noticeable effects on the continuance intention. Among them, the significance of assurance (β = 0.226, C.R. = 4.32, p < 0.001), entertainment (β = 0.274, C.R. = 5.90, p < 0.001), anthropomorphism (β = 0.227, C.R. = 4.95, p < 0.001), and tangibles (β = 0.149, C.R. = 3.56, p < 0.001) all proved the relevant hypothesis. This has indicated that compared to reliability, customers are more concerned about the performance of robots in providing professional services, interactive fun, humanized performance and exterior design. The entertainment had the most significant effects on the continuance intention, which demonstrated robots with entertainment functions can greatly enhance customers’ willingness to use. In summary, these results have stressed that in improving the quality of robot services, special attention should be paid to assurance, entertainment, anthropomorphic features and tangibles design to better meet customer needs and promote continuance intention.

**TABLE 4:** The impact of service quality on continuance intention.

Relationships	S.E	β	C.R	p value	Hypothesis	Decision
REL→CI	0.045	−0.009	−0.24	n.s	H1a	Rejected
ASS→CI	0.054	0.226	4.32	***	H1b	Supported
ENT→CI	0.044	0.274	5.90	***	H1c	Supported
ANTH→CI	0.038	0.227	4.95	***	H1d	Supported
TAN→CI	0.058	0.149	3,56	***	H1e	Supported

n.s = not supported, β = standardized regression weights, p = probability.

***p < 0.001.

### 4.2 Moderating role of personal innovation

The moderating effect of personal innovation on the relationship between the quality of robot service in hotels and continuance intention was analyzed, as exhibited in Table 5, by employing the PROCESS macro (Model 1) in SPSS with mean-centered variables. The results showed that the personal innovation significantly moderated the relation between assurance (β = 0.358, t = 3.088, p = 0.002), entertainment (β = 0.283, t = 2.673, p = 0.008), anthropomorphism (β = 0.423, t = 4.481, p = 0.000), tangibles (β = 0.887, t = 4.472, p = 0.000) and continuance intention. All the moderating effects were positive. It meant the increase of personal innovation can cause the relation between customers’ perception of these service quality dimensions and their continuance intention to become more significant. Specifically, among customers with high personal innovation, the impact of assurance, entertainment, anthropomorphism and tangibles on the continuance intention was more pronounced.

**TABLE 5:** The moderating effect of personal innovation.

Relationships	Interaction coefficient	t-statistics	p	LLCI	ULCI	Hypothesis	Decision
REL→CI	0.161	1.056	0.292	−0.139	0.462	H2a	Rejected
ASS→CI	0.358	3.088	0.002	0.130	0.586	H2b	Supported
ENT→CI	0.283	2.673	0.008	0.075	0.491	H2c	Supported
ANTH→CI	0.423	4.481	0.000	0.238	0.609	H2d	Supported
TAN→CI	0.887	4.472	0.000	0.497	1.277	H2e	Supported

**FIGURE 2:**
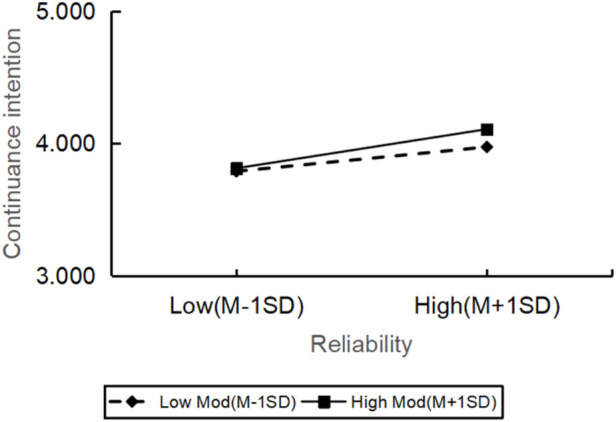
The moderating role of personal innovativeness on reliability and continuance intention.

**FIGURE 3:**
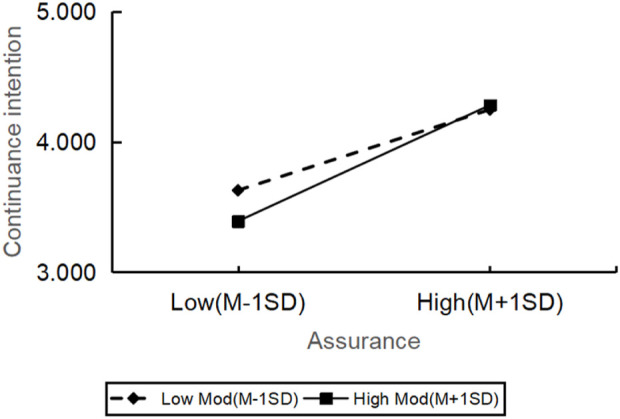
The moderating role of personal innovativeness on assurance and continuance intention.

**FIGURE 4:**
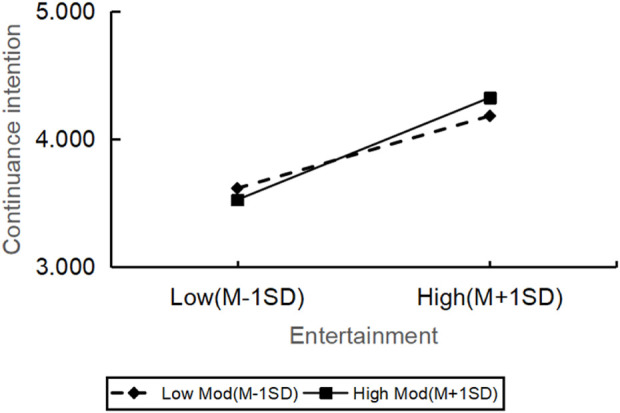
The moderating role of personal innovativeness on entertainment and continuance intention.

**FIGURE 5:**
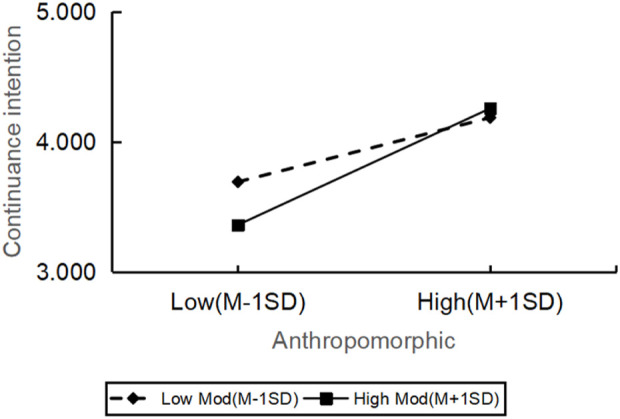
The moderating role of personal innovativeness on anthropomorphic and continuance intention.

**FIGURE 6:**
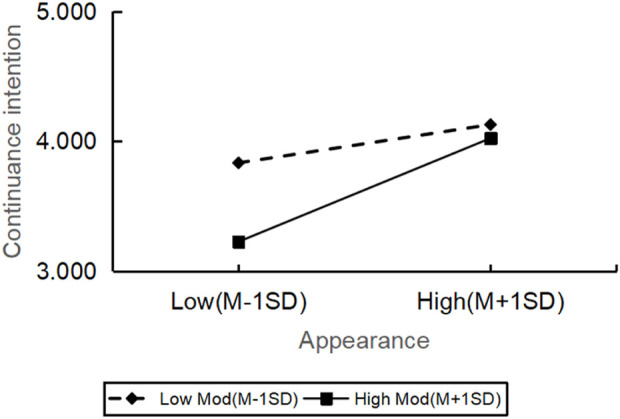
The moderating role of personal innovativeness on tangibles and continuance intention.

However, the relationship between reliability (β = −0.485, t = −0.735, p = 0.463) and continuance intention was not significantly moderated by personal innovation. All in all, there were significant differences in how the personal innovation affected the continuance intention across various dimensions of service quality, but the impact on reliability was not significant (Figures 2–6).

## 5 Conclusion

### 5.1 Theoretical contributions

This study offers several theoretical contributions to the literature on service quality in robotic hospitality and its impact on continuance intention, particularly among Generation Z consumers in China. First, while prior studies have primarily focused on general service quality frameworks or technology acceptance models (e.g., TAM, UTAUT), few have examined how specific service quality dimensions—such as anthropomorphism and entertainment—apply to AI-driven hotel robots. Existing research often treats service quality as a unidimensional or generalized construct, without fully capturing the unique characteristics of robotic services. This study addresses this gap by extending the SERVQUAL model to incorporate robot-specific attributes (i.e., entertainment and anthropomorphism), thereby offering a more contextually grounded and nuanced understanding of service quality in human-robot interaction.

Second, although there is growing interest in robotic services in hospitality, empirical research linking robot service quality to continuance intention remains scarce, especially for younger digital-native cohorts like Generation Z. This study contributes by providing empirical evidence on how each dimension of robot service quality (reliability, assurance, entertainment, anthropomorphism, and tangibles) influences users’ intention to continue using such services. This adds theoretical richness by highlighting differential effects of quality dimensions in a robotic context. Third, the study introduces personal innovativeness as a key moderating variable, shedding light on individual differences in technology adoption. While previous studies often overlook the role of personal traits in the service quality–behavioral intention relationship, this research demonstrates that individual-level innovation readiness can strengthen or weaken the effect of perceived robot service quality on continuance intention. This contributes to the theoretical integration of technology acceptance and consumer behavior theories in the context of robotic services.

Lastly, by focusing on AI-driven service robots in the hotel industry, this study advances theoretical knowledge in the emerging field of hospitality robotics. It offers a conceptual model that can serve as a foundation for future research on robot adoption, human-robot interaction, and service innovation in hospitality and beyond.

### 5.2 Managerial implications

The primary aim of this study is to empirically examine the relationship between the service quality of hotel robots and customers’ continuance intention, thereby offering foundational insights for developing effective marketing strategies and enhancing robot service quality to foster long-term customer engagement. Based on the findings, several practical and theoretical implications can be drawn for both the hotel and robotics industries.

First, this study specifically focuses on Generation Z customers in China who have experienced item delivery robots in hotel settings. The results underscore the importance of understanding how perceived service quality affects their intention to continue using such services. The analysis reveals that assurance, entertainment, anthropomorphism, and tangibles exert significant positive effects on continuance intention. When customers perceive delivery robots as trustworthy and stable, they tend to regard them as efficient and safe tools. Entertainment—defined as the enjoyment and fun experienced during interactions with robots—also plays a key role in enhancing satisfaction and user engagement. By offering interactive and engaging experiences, delivery robots evolve from simple service devices into experiential tools that customers actively enjoy using.

Second, reliability did not show a significant relationship with continuance intention. This may partly be explained by the “novelty effect,” where users initially prioritize engaging experiences over basic task performance. Moreover, concerns regarding data privacy and system security—especially when sensitive information is involved—could complicate perceptions of reliability. Therefore, hotel operators should not only ensure the technical reliability of robots but also actively communicate data protection and security measures transparently, building user trust alongside technological performance.

Third, the moderating role of personal innovativeness was confirmed, particularly for Generation Z, known for openness to technology and preference for personalized, interactive services. Practically, hotels should identify highly innovative customers through behavioral data analysis or targeted surveys and tailor robot experiences to their preferences. Offering early access to new features or customization options can enhance engagement and advocacy among this segment. Marketing campaigns highlighting novelty, creativity, and technological sophistication may effectively attract and retain these innovation-prone customers.

In summary, by strategically enhancing specific service quality dimensions and aligning offerings with users’ innovation readiness, hotel operators can not only increase customer retention but also position their brand as a pioneer in smart hospitality. This study offers a roadmap for leveraging service robots not just as operational tools, but as value-creating agents that shape long-term customer relationships and competitive advantage in the hospitality industry.

### 5.3 Limitation and further research directions

While this study offers valuable insights into the relationship between hotel service robot quality and customers’ continuance intention—particularly considering the moderating role of personal innovativeness—it is not without limitations, which provide avenues for future research.

First, the study’s sample is restricted to Generation Z consumers in China. Although this demographic represents a technologically adept and influential market segment, the findings may not be generalizable to older generations or consumers from different cultural or socioeconomic backgrounds. Generational cohorts may vary in their technology readiness, privacy concerns, or expectations regarding human-robot interaction. Therefore, future research should expand the sample to include a broader age spectrum and cross-cultural comparisons, exploring how generational and cultural variations affect perceptions of robotic service quality and continuance intention. Comparative studies across regions with different technological infrastructures and sociocultural attitudes toward automation would yield richer theoretical and practical implications.

Second, the data collection employed random sampling. Due to uneven development across Chinese cities, the adoption of robotic services varies by region. Respondents from technologically advanced areas have greater exposure to these services and tend to hold more positive evaluations, whereas those from less developed regions, with limited exposure, may exhibit skepticism or unfamiliarity. Future research should adopt stratified sampling to reflect regional differences in technology adoption rates, robot penetration, and user experience, thereby capturing a more comprehensive picture of consumer attitudes nationwide.

Third, this study employed a cross-sectional survey design, capturing consumer attitudes at a single point in time. However, continuance intention is inherently a longitudinal construct influenced by repeated usage, evolving trust, and experience-based learning. Future research should adopt longitudinal or experimental designs to observe how perceptions of service quality and continuance behavior evolve over time as consumers gain more familiarity with robotic services.

Finally, while the study integrated key dimensions of service quality and a personal innovativeness moderator, other potentially influential variables—such as technological anxiety, trust propensity, service failure experiences, or perceived value—were not included. Further research should explore these and other mediating or moderating mechanisms to enhance the explanatory power of the proposed model. In summary, addressing these limitations through more diverse sampling, longitudinal methods, and expanded theoretical models will deepen our understanding of how consumers engage with robotic services in hospitality and beyond.

## Data Availability

The raw data supporting the conclusions of this article will be made available by the authors, without undue reservation.
